# Mouse Hepatic Tumor Vascular Imaging by Experimental Selective Angiography

**DOI:** 10.1371/journal.pone.0131687

**Published:** 2015-07-01

**Authors:** Sang Kyum Kim, Honsoul Kim, Gou Young Koh, Dae-Sik Lim, Dae-Yeul Yu, Man Deuk Kim, Mi-Suk Park, Joon Seok Lim

**Affiliations:** 1 Department of Pathology, Yonsei University College of Medicine, Seoul, Republic of Korea; 2 Department of Radiology, Research Institute of Radiological Science, Yonsei University College of Medicine, Seoul, Republic of Korea; 3 National Research Laboratory of Vascular Biology and Stem Cell, Graduate School of Medical Science and Engineering, Korea Advanced Institute of Science and Technology (KAIST), Daejeon, Republic of Korea; 4 Department of Biological Sciences, Korea Advanced Institute of Science and Technology (KAIST), Daejeon, Republic of Korea; 5 Aging Research Center, Korea Research Institute of Bioscience and Biotechnology, Daejeon, Republic of Korea; The University of Hong Kong, CHINA

## Abstract

**Purpose:**

Human hepatocellular carcinoma (HCC) has unique vascular features, which require selective imaging of hepatic arterial perfusion and portal venous perfusion with vascular catheterization for sufficient evaluation. Unlike in humans, vessels in mice are too small to catheterize, and the importance of separately imaging the feeding vessels of tumors is frequently overlooked in hepatic tumor models. The purpose of this study was to perform selective latex angiography in several mouse liver tumor models and assess their suitability.

**Materials and Methods:**

In several ectopic (Lewis lung carcinoma, B16/F10 melanoma cell lines) and spontaneous liver tumor (*Albumin-Cre/MST1^fl/fl^/MST2^fl/fl^*, *Albumin-Cre/WW45^fl/fl^*, and *H-ras12V* genetically modified mouse) models, the heart left ventricle and/or main portal vein of mice was punctured, and latex dye was infused to achieve selective latex arteriography and/or portography.

**Results:**

*H-ras12V* transgenic mice (a HCC and hepatic adenoma model) developed multiple liver nodules that displayed three different perfusion patterns (portal venous or hepatic artery perfusion predominant, mixed perfusion), indicating intra-tumoral vascular heterogeneity. Selective latex angiography revealed that the Lewis lung carcinoma implant model and the *Albumin-Cre/WW45^fl/fl^* model reproduced conventional angiography findings of human HCC. Specifically, these mice developed tumors with abundant feeding arteries but no portal venous perfusion.

**Conclusion:**

Different hepatic tumor models showed different tumor vessel characteristics that influence the suitability of the model and that should be considered when designing translational experiments. Selective latex angiography applied to certain mouse tumor models (both ectopic and spontaneous) closely simulated typical characteristics of human HCC vascular imaging.

## Introduction

The liver is unique in that it receives dual input perfusion from both an arterial and venous supply. The liver parenchyma is predominantly perfused by portal venous blood and to a lesser extent by hepatic arterial blood. However, as hepatocarcinogenesis occurs during the development of hepatocellular carcinoma (HCC), perfusion from aberrant arterioles gradually replaces the normal dual perfusion from the paired vessels [1–3]. As a result, HCC has unique but complex hemodynamic features, which are not only biologically meaningful but also serve as the basis for its routine radiological diagnosis and treatment. HCC early arterial phase enhancement and delayed phase washout are typical findings on contrast-enhanced dynamic imaging studies [4, 5]. Meanwhile, such dual input perfusion of the liver and the complex hemodynamics of HCC indicate the need for separately evaluating the hepatic arterial and portal venous circulation. In humans, this challenging task can be accomplished by selectively catheterizing a vessel of interest to physically isolate the blood perfusion it conveys, followed by angiography. More delicate methods beyond conventional angiography, such as computed tomography during arterial portography and computed tomography during hepatic arteriography, are also available and can separately image the distribution of the intra-hepatic portal and arterial blood flow with high contrast resolution [6]. Based on these methods, clinical research has expanded our knowledge in this field and revealed that the intranodular blood supply is altered in parallel with the process of hepatocarcinogenesis from a dysplastic nodule to overt HCC [2, 7, 8]. However, these bedside observations have rarely been expanded to the bench with the goal of understanding the underlying pathophysiology of such hemodynamic events.

Compared to angiography in humans, performing selective angiography in small animals is extremely challenging [9] because the vasculature of small animals is simply too small for the insertion of vascular catheters. Significant progress has been achieved in experimental microvascular imaging methods of small animals in organs other than the liver [10, 11], but unfortunately, these methods are often not optimal for the imaging of hepatic tumor feeding vessels if the intention of the study design pertains to the concept of the aforementioned dual input perfusion of the liver and HCC hemodynamics. As a result, selective imaging of the tumor-feeding vessel is rarely performed in mouse hepatic tumor models, and the suitability as an experimental platform is frequently overlooked, even in translational studies that focus on HCC hemodynamics and/or radiological aspects. We envisioned that a selective angiography method that is applicable to mice would be useful for characterizing the tumor feeding vessels in hepatic tumor models and assessing their suitability as an experimental platform, especially when hemodynamic aspects are emphasized or radiological translational research is involved. In this study, we adopted a commercially available latex dye used for vascular biology experiments and applied the vascular cast method [12, 13] to perform selective vascular imaging of mouse liver tumors.

The purpose of this study was to examine the tumor feeding vessels in several experimental mouse hepatic tumor models using selective latex angiography to identify mouse tumor models that resemble the vascular (or radiological) characteristics of human HCC.

## Materials and Methods

### Mice

The Animal Care Committee of Severance Hospital (Permit number: 2014–0199) and Korea Advanced Institute of Science and Technology (KAIST, Permit number: KA2011-28) approved this study. Specific pathogen-free C57BL/6J mice were purchased from Jackson Laboratory and bred in our pathogen-free animal facility. All animals were fed a standard normal diet (PMI Lab Diet, St. Louis, MO) *ad libitum* with free access to water. Eight- to ten-week-old male mice were used for this study, unless otherwise specifically indicated.

### Ectopic liver tumor models

Mouse Lewis lung carcinoma (LLC, Catalog number: CRL-1642) and B16/F10 melanoma (Catalog number: CRL-6475) cell lines were obtained from the American Type Culture Collection. The Hepa1c1c7 HCC (Catalog number: 22026) cell line was obtained from the Korean Cell Line Bank (Seoul, Korea). The background of these cell lines is syngeneic to C57BL/6J mice.

Mice were anesthetized by intramuscular injection of a mixture of anesthetics (80 mg/kg ketamine and 12 mg/kg xylazine). Skin and peritoneal incisions were made at the midline of the upper abdomen to expose the left lateral lobe of the liver. An *in vitro* cultured cell line suspension (2.5 × 10^5^ cells in 20 μl of cooled PBS) was loaded into a 20-gauge insulin syringe, and subcapsular implantation was performed in the left lateral lobe of the liver (LLC implantation: n = 20, B16/F10 implantation: n = 9, and Hepa1c1c7 implantation: n = 25). The incision wound was closed with 4–0 black silk sutures.

For the Hepa1c1c7 cell line, implantation of 2.5 × 10^5^ cells (n = 10) did not produce a hepatic mass. We repeated implantation with 1 × 10^6^ (n = 10) and 5 × 10^6^ cells (n = 5) and waited up to 3 months before sacrificing the mice; however, no liver tumors were detected.

### Spontaneous hepatic tumor models

Three types of spontaneous hepatic tumor models were used. The first two models were *Albumin-Cre/MST1* ^*fl/fl*^ */MST2* ^*fl/fl*^ (liver-specific *MST1* and *MST2* knockout) mice and *Albumin-Cre/WW45* ^*fl/fl*^ (liver-specific *WW45* knockout) mice, which spontaneously develop HCC [14] and HCC with an intermediate phenotype [15], respectively. Both types of mice were kind gifts from Professor Dae Sik Lim at KAIST. *MST1* ^*fl/fl*^ */MST2* ^*fl/fl*^ and *WW45* ^*fl/fl*^ mice were each crossed with *Albumin-Cre* transgenic mice, which express Cre recombinase in the postnatal liver. Genotyping of the mice was performed using genomic DNA obtained from samples of tail biopsies.

Genotyping of *MST1* was done by polymerase chain reaction (PCR) analysis with forward primer 5′-GTGGATCTTTCCGTTTTTGG-3′ and reverse primer 5′-CCAAAGGCAACGATAAA-3′, which yielded products of 500 bp and 300 bp for the undeleted and excised alleles, respectively. Genotyping of *MST2* was conducted by PCR analysis with forward primer 5′-AAAGCTGGTACTGGGGTTCA-3′ and reverse primer 5′-CCCTAACCCCCATTGAACTT-3′, which yielded products of 800 bp and 500 bp for the undeleted and excised alleles, respectively. Genotyping of *WW45* was performed by PCR with forward primer 5′-CTTATGCCCTTTTGTTTGAT-3′ and reverse primer 5′-TGCTGGTTTTGTCTCACTAA-3′, which yielded products of 1,333 bp and 165 bp for the undeleted and excised alleles, respectively [15]. Male and female mice aged 10 to 12 months were used, as specified. The hepatic tumors harvested from *Albumin-Cre/MST1* ^*fl/fl*^ */MST2* ^*fl/fl*^ (n = 3) and *Albumin-Cre/WW45* ^*fl/fl*^ (n = 3) were fixed with 10% formalin. Hematoxylin and eosin staining was performed for histologic analysis.

The third type of spontaneous hepatic tumor model, *H-ras12V* transgenic mice were a kind gift from Professor Dae-Yeul Yu at Korea Research Institute of Bioscience and Biotechnology. Transgenic lines of mice were established by mating these mice with C57BL/6J mice. Genotyping was performed by PCR with forward primer 5′-CTAGGGCTGCAGGAATTC-3′ and reverse primer 5′-GTAGTTTAACACATTATACACT-3′. Transgenic mice yielded a product of 711 bp [16]. Here, 10-month-old male mice were used.

### Example images of conventional angiography of human HCC

Our institutional image depository, based on a picture archiving and communicating system, was randomly searched to find a representative human HCC image demonstrating typical conventional angiographic findings. This image was used as a side-by-side display to describe the similarities between human conventional angiography and mouse selective angiography. No medical information from the patient (other than the diagnosis of HCC) was obtained.

### Selective angiography of the hepatic vessels

Anesthesia was achieved by intramuscular injection of a mixture of anesthetics (80 mg/kg ketamine and 12 mg/kg xylazine). Systemic arteriography was performed as previously described [17]. Briefly, the anterior side of the chest was opened, and an outflow opening was made at the right atrium of the beating heart. The left ventricle of the heart was punctured with a blunted 18-gauge needle, and vascular latex dye (Connecticut Valley Biological Supply Co., Southampton, MA) or acryl paint (Alpha Colors, Seoul, Korea) was slowly and gently injected with a 10-ml syringe. To achieve portography [13] and venography, a midline incision was made, and the colon and small bowel were shifted to the left to expose the target vessels. Then, the main portal trunk (for portography) or the inferior vena cava (for venography) was punctured with a 22-gauge angiocatheter for latex dye infusion. An outflow opening was made at either the right atrium of the heart (for portography) or main portal vein (for venography). Either cannulation failure or extravascular leakage from an unsecure puncture site resulting in suboptimal latex dye infusion was considered as a technical failure.

For dual angiography, arteriography and portography were sequentially performed with different color dyes in the same mouse. First, under anesthesia, an angiocatheter was inserted into the main portal vein. Next, arteriography with blue latex dye was performed as described above. Immediately afterwards, portography with green acryl paint was conducted via the pre-inserted porto-venous angiocatheter.

After angiography, the liver was resected, briefly washed in phosphate buffered saline, and gross images were obtained. Then, the specimen was fixed with 10% formalin overnight. For whole-mount imaging, a tissue clearance procedure was performed to enhance sample transparency and maximize delineation of the vascular casts. The specimen was dehydrated by processing through a methanol series and cleared by immersion in a mixture of organic solvents (benzyl alcohol/benzyl benzoate, 1:1 [Sigma-Aldrich, Saint Louis, MO]) [13].

## Results

### Selective angiography in mice with vascular catheter insertion and latex dye infusion

**Fig 1:**
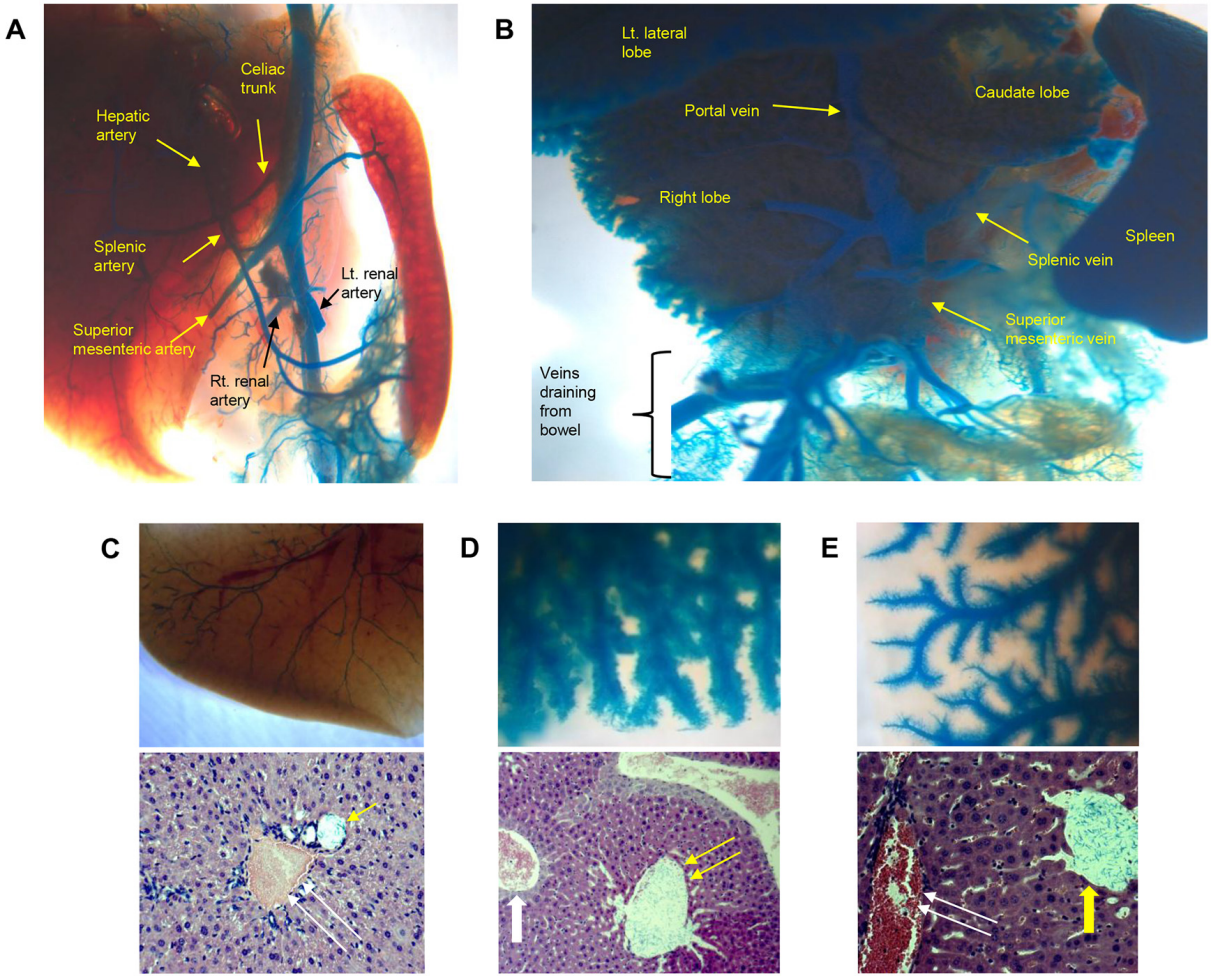
Selective angiography using blue latex dye. (A) Systemic arteriography and (B) direct portography in a wild-type mouse. The stomach, duodenum, and jejunum were removed to allow a clear view of the vessels. (C-E) Magnified images of the liver after selective angiography and subsequent tissue clearing procedures (upper panels) and histological assessment (lower panels, hematoxylin-eosin staining) were performed. (C) Arteriography, (D) portography, and (E) hepatic venography specimens are shown. Blue latex dye particles are seen only in the target vascular compartment (yellow arrows), which seems to have been slightly dilated during injection (single arrow, hepatic artery; double arrow, portal vein; thick arrow, central vein). Blue latex dye particles are not detectable in non-target vascular compartments (white arrows).

Latex dye infusion was successfully utilized for selective arteriography and portography in adult C57BL/6J mice. On arteriography, the abdominal aorta, celiac trunk, common hepatic artery, splenic artery, renal arteries, and intra-hepatic arterial structures were clearly visible (Fig 1A). Portography produced dense latex perfusion of the whole liver and visualization of the main portal vein, splenic vein, and superior mesenteric vein (Fig 1B). Magnified images of the liver after tissue clearance procedures clearly delineated the fine structures of the intra-hepatic vessels (Fig 1C–1E, upper panels). Microscopic examination revealed that passage of the latex dye into the hepatic sinusoid was minimal and that the majority of the perfused latex dye remained in each specific target vascular compartment (Fig 1C–1E, lower panels).

### Tumor feeding vessels can receive blood from both the hepatic artery and portal vein

To test whether selective latex angiography can be used to visualize vascular heterogeneity in a manner similar to what is seen in human hepatocarcinogenesis, a tumor model with heterogeneous nodules was required. Therefore, we used 10-month-old *H-ras12V* over-expressing transgenic mice. These mice develop multi-centric spontaneous hepatic tumors consisting of both hepatic adenoma and HCC [18], and therefore, the nodules produced in this model are heterogeneous. Therefore, we envisioned that simultaneous arterial and portal selective angiography might be necessary. We performed dual angiography using six *H-ras12V* transgenic mice (Table 1), but procedure failure occurred in half (n = 3) of them, and only three mice were successful. The high failure rate was due to the difficulty of performing cardiac puncture and portal venous cannulation in the same mouse.

**Table 1:** Summary of the angiography procedures performed in each tumor model.

Model	Arteriography	Portography
LLC implanted mice	Performed	Performed
B16/F10 melanoma implanted mice	Performed	Not performed
Hepa1c1c7 hepatocellular carcinoma implanted mice	Not performed	Not performed
*Albumin-Cre/MST1* ^*fl/fl*^ */MST2* ^*fl/fl*^ mice	Performed	Not performed
*Albumin-Cre/WW45* ^*fl/fl*^ mice	Performed	Performed
*H-ras12V* transgenic mice	Dual angiography	

**Fig 2:**
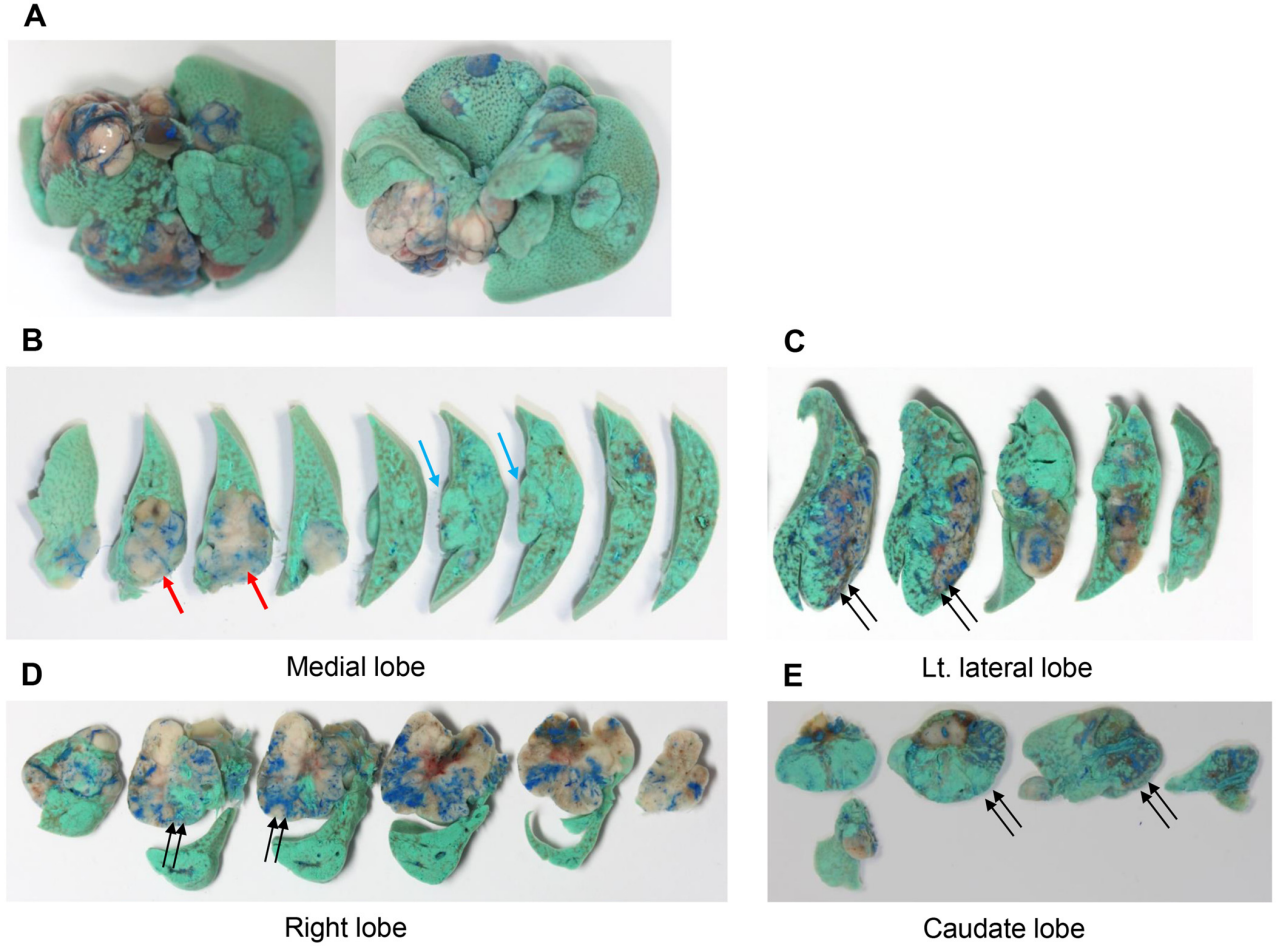
Liver dual angiography performed in a *H-ras12V* over-expressing transgenic mouse that developed multiple spontaneous tumors. Selective latex arteriography (blue latex) and portography (green acrylic paint) were sequentially conducted. Gross images of (A) the whole liver superior aspect (left) and inferior aspect (right), and sectioned slices of (B) the medial lobe, (C) left lateral lobe, (D) right lobe, and (E) caudate lobe. Nodules were perfused by arterial supply (red arrow), portal venous supply (blue arrow), or both (double arrows).

We were able to observe 14 hepatic nodules which demonstrated three different vascular perfusion patterns: 1) blue latex only (hepatic arterial perfusion predominant; 2 nodules), 2) green acryl only (portal venous perfusion predominant; 4 nodules), and 3) mixed (hepatic arterial and portal venous dual perfusion; 8 nodules) (Fig 2).

### The perfusion profile of liver-implanted LLC resembled that of human HCC

Selective latex arteriography was attempted in 20 mice (LLC; n = 11, B16/F10; n = 9). Three procedure failures occurred during selective latex arteriography (LLC; n = 2, B16/F10; n = 1). Selective latex portography was attempted in 9 mice (LLC; n = 9, B16/F10; n = 0). Selective latex portography in B16/F10 melanoma implanted mice was not performed because we believed that their arteriography feature was not satisfactory. One procedure failure occurred during selective latex portography using LLC-implanted mice. Consequently, selective latex arteriography was successfully performed in 17 cell line-implanted mice (LLC; n = 9, B16/F10; n = 8), whereas selective latex portography was achieved in eight mice (LLC; n = 8, B16/F10; n = 0), (Table 1, S1 File).

**Fig 3:**
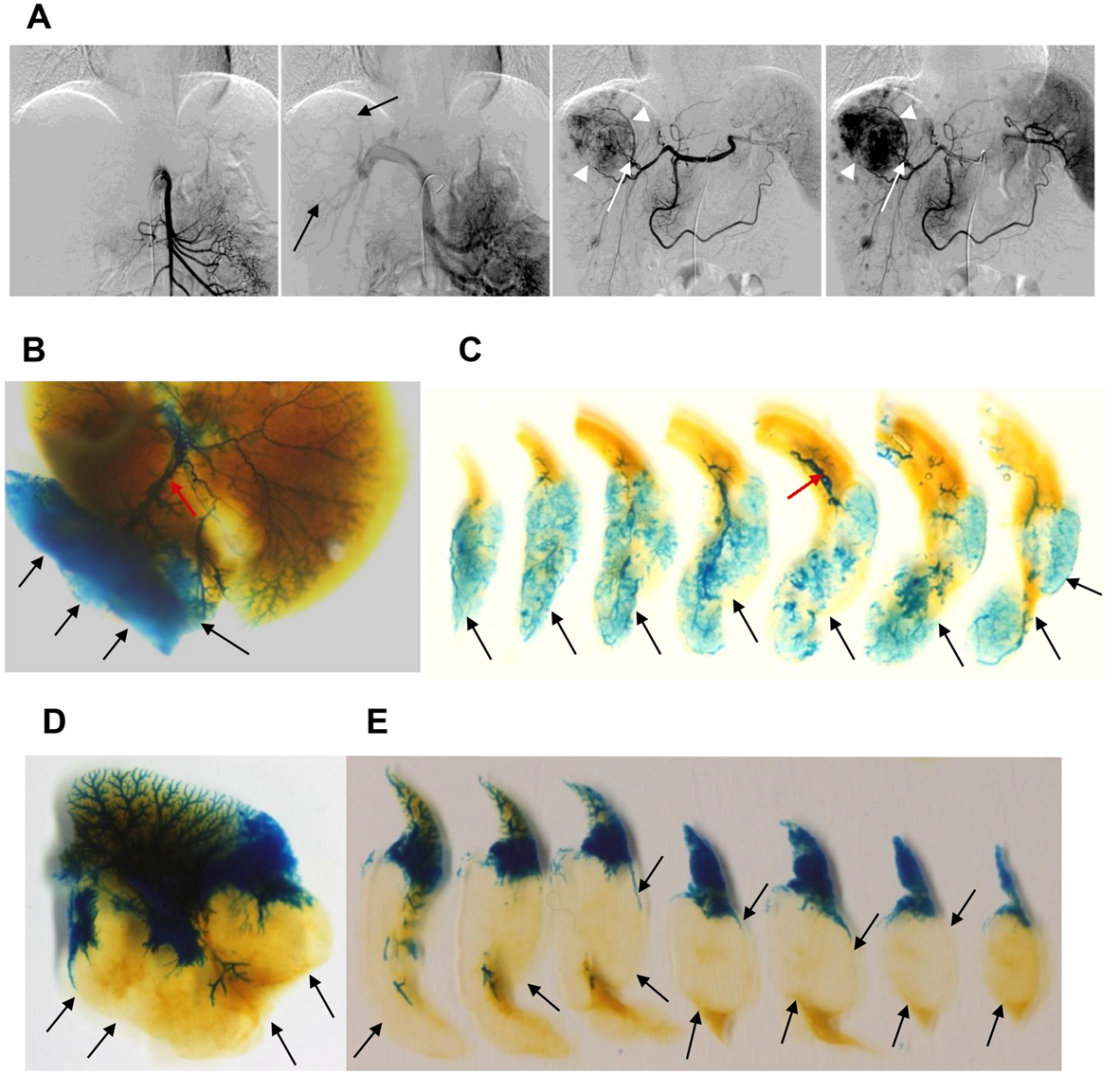
Conventional angiography of human hepatocellular carcinoma and latex angiography in mouse Lewis lung carcinoma model. (A) Representative images of conventional angiography of a typical case of human hepatocellular carcinoma. From left, superior mesenteric arterial angiography, indirect portography, celiac trunk arterial angiography (early phase), and celiac trunk arterial angiography (late phase) images are shown. The branches of the tumor-feeding artery supplying the medial portion of the mass (white arrow) and displaced portal veins (black arrow) are clearly visible. Note that the main mass (white arrowheads) and the multiple small satellite nodules are extremely hypervascular in nature. (B-E) Selective latex angiography of a liver Lewis lung carcinoma ectopic tumor model. (B-C) Selective latex arteriography and the subsequent tissue clearance procedure were performed in a liver Lewis lung carcinoma-bearing mouse. (B) Gross image of the whole liver and (C) sectioned slices demonstrate the hypervascular mass (arrows) supplied by the hepatic artery. The enlarged, slightly tortuous tumor-feeding artery (red arrow) is clearly visible. (D-E) Selective latex portography and the subsequent tissue clearance procedure were performed in a liver Lewis lung carcinoma-bearing mouse. (D) Gross image of the whole liver and (E) sectioned slices show that portal venules do not enter the mass (arrows).

Human HCC typically receives a rich hepatic artery blood supply but is devoid of a portal venous blood supply [8, 19] (Fig 3A). Selective arteriography of LLC-implanted tumors revealed a markedly hypervascular mass supplied by tumor feeding vessels originating from the hepatic artery (n = 9, Fig 3B and 3C). Meanwhile, portography demonstrated only minimal latex dye staining that was confined to the periphery of the mass; the mass center was not perfused (n = 8, Fig 3D and 3E). Such angiographic features resemble those of conventional angiography findings in human HCC (Fig 3A).

Because human melanoma liver metastases frequently present as hypervascular masses [20], we performed selective latex arteriography using a B16/F10 melanoma liver implant model (n = 8). However, the B16/F10 ectopic tumor model did not demonstrate a conclusive perfusion profile as the vascularity of the mass revealed by arteriography was not considerably prominent. Another technical obstacle was that B16/F10 melanoma cells produced abundant black melanin pigments which interfered with visual assessment of the intratumoral vessel structures. Therefore, we did not proceed with latex portography. We also tested a Hepa1c1c7 HCC implant model, which was based on a cell line derived from a syngeneic background; however, no tumor masses were generated (n = 25).

### Selective angiography in spontaneous hepatic tumor models

Selective latex arteriography was performed using four *Albumin-Cre/MST1* ^*fl/fl*^ */MST2* ^*fl/fl*^ mice, and no procedure failures occurred. Selective portography was not performed using these mice because we considered the arteriography features of their background liver not satisfactory. In *Albumin-Cre/WW45* ^*fl/fl*^ mice, selective latex arteriography and portography were performed in four mice for each procedure. One procedure failure occurred each for selective latex arteriography and portography. Therefore, selective latex arteriography was successfully achieved in seven genetically modified mice with spontaneous tumors (*Albumin-Cre/MST1* ^*fl/fl*^ */MST2* ^*fl/fl*^; n = 4, *Albumin-Cre/WW45* ^*fl/fl*^; n = 3), whereas selective latex portography was successfully performed in three mice (*Albumin-Cre/MST1* ^*fl/fl*^ */MST2* ^*fl/fl*^; n = 0, *Albumin-Cre/WW45* ^*fl/fl*^; n = 3), (Table 1, S1 File).

**Fig 4:**
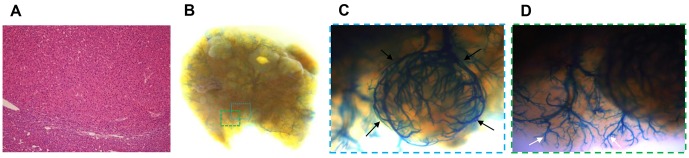
Selective latex arteriography of an *Albumin-Cre/MST1* ^*fl/fl*^ */MST2* ^*fl/fl*^ mouse that developed multiple spontaneous hepatocellular carcinomas. (A) Histologic examination (hematoxylin and eosin staining, ×100). (B) The whole liver and (C, D) magnified images from a surgical microscope display a hypervascular mass supplied by feeding arteries (black arrow) and enlarged prominent arteries (white arrow) in the tumor-free background liver. The dotted box in (B) indicates the region magnified in (C, D).

We performed selective latex arteriography (n = 4) in 10-month-old genetically modified *Albumin-Cre/MST1* ^*fl/fl*^ */MST2* ^*fl/f l*^ mice, which are a model of spontaneous HCC (Fig 4A), [14]. These mice produced numerous liver nodules that demonstrated abundant tumor feeding arteries (Fig 4B and 4C). Relatively abundant tortuous prominent arteries appeared in the tumor-free liver as well (Fig 4D), which caused the background liver to appear somewhat noisy and therefore we determined this model not suitable for latex angiography.

**Fig 5:**
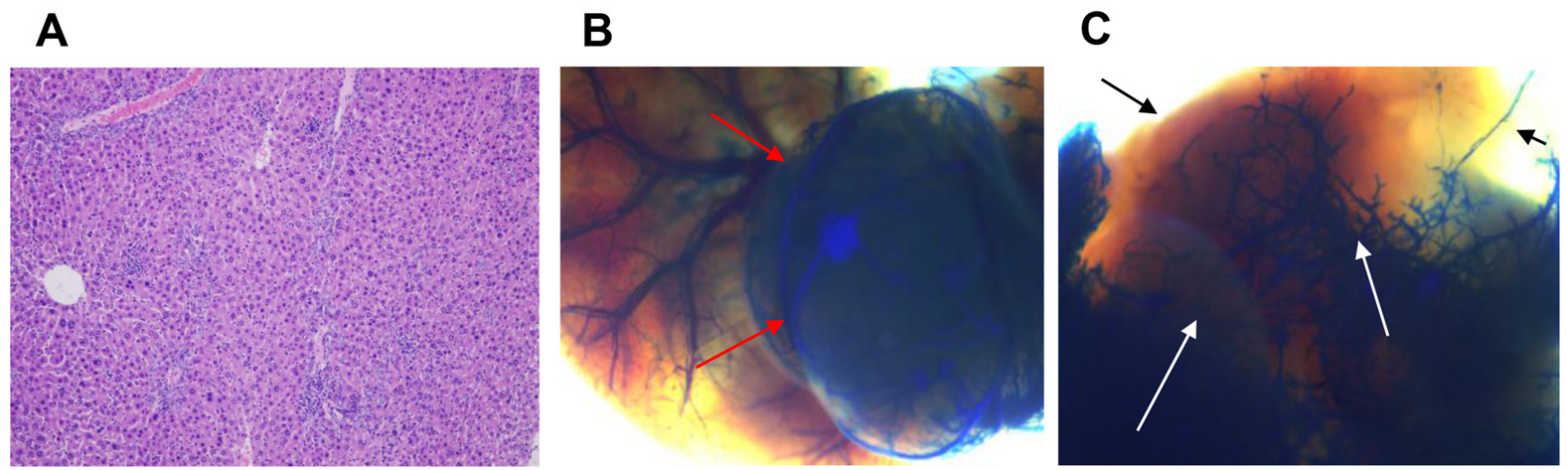
Latex angiography of *Albumin-Cre/WW45* ^*fl/fl*^ mice that developed spontaneous hepatocelluar carcinomas. Representative images of (A) histologic examination (hematoxylin and eosin staining, ×100), (B) selective latex arteriography and (C) portography. The masses appeared as extremely hypervascular lesions (red arrows) on selective latex arteriography, but were devoid of portal venous perfusion (black/white arrows).

We conducted selective latex angiography in 10-month-old *Albumin-Cre/WW45* ^*fl/fl*^ mice, which develop HCC (Fig 5A), [15]. These mice produced multiple liver nodules, which were rich in feeding arteries when selective latex arteriography (n = 3) was performed (Fig 5B). Unlike in the *Albumin-Cre/MST1* ^*fl/fl*^ */MST2* ^*fl/fl*^ model, arteries in the tumor-free background liver were not increased, but rather they remained stable (Fig 5B), showing an appearance similar to that of wild-type mice (Fig 1C). Selective latex portography (n = 3) demonstrated that these nodules did not receive significant portal venous perfusion (Fig 5C).

## Discussion

Unlike in the clinical field where HCC hemodynamics has been extensively studied, the concept of vessels that feed liver tumors and the associated hemodynamics remain somewhat unfamiliar in the domain of basic research involving tumor biology. However, we believe that extensive basic research in this field is indispensable for performing mechanistic dissections and elucidating the underlying pathogenesis. Moreover, the hemodynamic characteristics have often been ignored when selecting a particular mouse tumor model for preclinical translational studies, which we believe may sometimes result in suboptimal simulation of the intended clinical setting. We proposed that the small size of mouse vessels is the major technical obstacle for selectively imaging liver tumor vasculature, because unlike human vessels, catheterization and thus selective angiography are impossible. To solve this problem, we used a commercially available vascular latex dye, which does not readily cross the capillary bed, to perform selective arteriography and portography in mice [13, 17]. Because liver sinusoid capillaries lie between hepatic arterioles, portal venules, and hepatic venules, we were able to selectively fill and visualize a target vascular compartment as long as an appropriate infusion inlet could be secured. This approach allowed us to avoid the need to catheterize the small-sized vessels, but to still successfully isolate a target vascular compartment from the rest of the systemic circulation and to selectively image the specific vasculature. Latex dye angiography was capable of vascular imaging with a field of view large enough to include the entire liver, but lacked the capability to visualize capillary size small vessels. In this sense it is similar with the digital microangiography method using barium particles [21, 22]. In contrast, this approach differs from optics based techniques widely applied for microangiography of small animals [23], which provide with excellent resolution at the cost of superficial imaging depth and narrow field of view.

Theoretically, a wide spectrum exists with a portal venous perfusion-dominant healthy liver at one end and an exclusively arterial-perfused high-grade HCC at the other end.

We sought to determine if such intra-tumoral vascular heterogeneity can be recapitulated in an experimental environment, and thus, we adopted the *H-ras12V* transgenic mouse model. These mice spontaneously develop multicentric HCC and hepatic adenoma within the same individual liver [18]. Therefore, we assumed that multiple, heterogeneous nodules would develop. We performed dual-selective angiography in these mice and observed three different perfusion patterns: 1) portal venous perfusion predominant, 2) mixed perfusion (both portal venous and hepatic arterial), and 3) hepatic arterial perfusion predominant. We believe that the presence of nodules showing different patterns of vessels supplying the tumors reflects the natural intra-tumoral vascular heterogeneity. Consequently, we concluded that our selective latex angiography method is capable of visualizing and differentiating different patterns of tumor vascular supply that are observed during the multi-step hepatocarcinogenesis in humans [2, 24, 25].

Based on selective latex angiography, we searched for a mouse liver tumor model with similar vascular imaging characteristics as human HCC. In the healthy liver, dual perfusion by the hepatic artery and portal vein feeds the liver, but the portal venous perfusion is predominant [2]. However, as hepatocarcinogenesis proceeds, histologically unpaired arterioles increase and radiological arterial perfusion dominates [2, 3, 24]. Eventually, an overt HCC is deprived of portal venous perfusion and receives its entire perfusion from aberrant arterioles [2, 26, 27]. Therefore, we searched for a tumor model in which the mass receives its blood supply solely from the hepatic artery and in which the background liver maintains predominant portal venous perfusion. We tested implantation of a few types of cell lines to create ectopic liver tumor models and found that the LLC-implanted ectopic liver tumor model produced a markedly hypervascular mass. The tumor developed abundant feeding arteries but lacked portal venous perfusion. These selective latex angiography findings resembled the typical features of human HCC revealed by radiological studies such as conventional angiography (Fig 3A). Meanwhile, the selective latex angiographic findings of the B16/F10 melanoma cell line-implanted liver tumor model were not impressive because the vascularity was not clear. The presence of the dense black melanin pigmentation caused another technical problem that interfered with clear visualization of the latex dye-infused vessels.

Next, we tested spontaneous hepatic tumor models of genetically modified mice. *Albumin-Cre/MST1* ^*fl/fl*^ */MST2* ^*fl/fl*^ mice are an HCC model [14] that develops hypervascular masses with rich tumor-supplying arteries. However, we did not proceed further with this model because the background liver also showed unexpectedly prominent arteries, which we determined were inappropriate for precise hemodynamic studies. *Albumin-Cre/WW45* ^*fl/fl*^ mice are a model of HCC with an intermediate phenotype [15]. The nodules in these mice resembled human HCC in that we observed rich tumor-feeding arteries but not significant portal venules that entered the internal portion of the tumor. Collectively, among the tumor models we tested, the LLC-implanted ectopic liver tumor and *Albumin-Cre/WW45* ^*fl/fl*^ mice seem to be models that most closely resemble the hemodynamic features of human HCC and are therefore appropriate for hemodynamic and/or radiological translational studies.

We recognize several limitations in our study. Most importantly, the selective latex angiography we describe is an *ex vivo* approach that does not allow serial assessments. Second, we acknowledge that the pathogenesis of our tumor models is different from that of human HCC. However, model suitability is a common issue encountered during experimental research, and therefore, we believe that our demonstrated angiographic similarity with human HCC is meaningful. Third, in the current study, we did not investigate the underlying pathogenesis that induced the different imaging patterns of hepatic tumor vessels we described. We believe that a biological mechanistic examination is beyond the scope of this study and should be elucidated in subsequent research studies.

In conclusion, selective latex angiography permitted selective vascular imaging in mouse hepatic tumor models. Based on this method, we demonstrated that the imaging findings of the LLC liver implantation model and selected genetically modified spontaneous hepatic tumor models resemble the radiological imaging findings of human HCC. We believe that selective latex angiography in combination with appropriate hepatic tumor models will serve as an optimized experimental platform that simulates the vessels that feed the tumors and/or the radiological features of human hepatic malignancies.

## Supporting Information

S1 File Summary of mouse chart, tumor model, procedure and observation. (XLSX)
